# Predictors of difficult vascular access during transradial neurointervention: a retrospective study

**DOI:** 10.1590/1806-9282.20260021

**Published:** 2026-06-22

**Authors:** Jian Wu, Fei Zhang, Quiping Li, Biao Qi

**Affiliations:** 1 Fudan University, Xiamen Branch, Zhongshan Hospital, Department of Neurosurgery – Xiamen, China.; 2 Fudan University, Xiamen Branch, Zhongshan Hospital, Department of Gynecology – Xiamen, China.

**Keywords:** Radial artery, Endovascular procedures, Vascular access, Catheterization, Brachiocephalic trunk, Internal carotid artery

## Abstract

**OBJECTIVE:**

The aim of this study was to identify factors associated with difficulty in establishing vascular access during the transradial approach for neurointerventional therapy.

**METHODS:**

We retrospectively analyzed consecutive patients who successfully underwent transradial approach neurointerventional therapy at Fudan Zhongshan Xiamen Hospital (July 2021–July 2024). Patients were categorized as "difficult" or "easy" according to whether vascular access establishment required >15 min. Collected variables included difficulty in radial artery puncture/sheath insertion, feasibility of routinely forming a Simmons-2 catheter, aortic arch type (II–III), tortuosity of the brachiocephalic trunk, brachiocephalic trunk–target common carotid artery angle (<30°), and tortuosity of the C2–C4 internal carotid artery. Multivariate logistic regression was performed to identify independent factors.

**RESULTS:**

Among 277 patients, 199 were in the easy group and 78 were in the difficult group. Compared with the easy group, the difficult group more frequently had difficult radial puncture/sheath insertion, brachiocephalic trunk tortuosity, a sharp brachiocephalic trunk–target common carotid artery angle (<30°), C2–C4 internal carotid artery tortuosity, and failure to routinely form a Simmons-2 catheter (all p<0.05). Hypertension, diabetes, hyperlipidemia, and type III aortic arch were not significantly different between groups (all p>0.05). On multivariate analysis, difficult radial puncture/sheath insertion, inability to routinely form a Simmons-2 catheter, brachiocephalic trunk tortuosity, a brachiocephalic trunk–target common carotid artery angle <30°, and C2–C4 internal carotid artery tortuosity were independent factors associated with access difficulty (all p<0.001).

**CONCLUSION:**

Both access-site difficulty and unfavorable supra-aortic/internal carotid artery anatomy were independently associated with prolonged vascular access establishment during transradial approach neurointerventional therapy.

## INTRODUCTION

Transradial access (TRA) has gained increasing acceptance in neurointerventional therapy and has been reported to achieve procedural success rates comparable to transfemoral access (TFA)^1^. Beyond feasibility, TRA is often favored because it may improve patient comfort and enable earlier mobilization, while potentially reducing access-site morbidity. However, despite these advantages, TRA has not replaced TFA as the default route in neurointervention. A major barrier is the frequent difficulty in establishing stable vascular access, particularly when treating anterior circulation lesions that require reliable support for device delivery and precise catheter control^2^.

In clinical practice, access difficulty during TRA can occur at multiple steps. At the puncture stage, repeated attempts and prolonged sheath insertion may increase procedure time and predispose to vasospasm, ultimately compromising the efficiency of the entire workflow. After successful sheath placement, constructing a stable pathway for selective catheterization may be limited by unfavorable supra-aortic anatomy, including challenges in routinely forming the Simmons-2 configuration and navigating tortuous or sharply angulated segments. Such technical resistance may lead to catheter kinking or herniation, reduced stability of the intermediate catheter, and increased fluoroscopy exposure, which can translate into higher procedural complexity and potentially higher risk.

Although multiple studies have described outcomes and technical considerations of TRA, practical evidence that quantifies which peri-access and anatomical features most strongly predict "difficult" access construction in real-world neurointervention remains limited. In addition, standardized, experience-based troubleshooting strategies—such as coaxial use of intermediate catheters to enhance support—are not always integrated into preprocedural planning.

Therefore, we retrospectively analyzed consecutive patients who successfully underwent TRA neurointerventional therapy at our institution and evaluated factors related to access difficulty. We defined difficult vascular access establishment as requiring more than 15 min and compared clinical and anatomical variables between "easy" and "difficult" cases. By identifying independent predictors of difficulty, we aimed to provide evidence-based suggestions to optimize patient selection, anticipate technical challenges, and improve the efficiency and safety of TRA in neurointerventional practice.

## METHODS

### Study subjects

Patients who underwent TRA neurointerventional therapy at Fudan University Affiliated Zhongshan Hospital Xiamen Hospital from July 2021 to July 2024 were included. Age ranged from 18 to 78 years. The neurointerventional therapies were all independently performed by the same team. The lead surgeon has 15 years of neurointerventional experience, which minimized potential biases or variations caused by the operator's experience.

Inclusion criteria: 1. Before radial artery puncture, palpable vascular pulsation was detected by palpation, and vascular diameter was assessed by ultrasound (radial artery diameter at least greater than 2.0 mm). 2. Presence of distal radial artery pulsation and the Allen's test is normal. 3. All patients had intracranial vascular diseases (above the C4 segment of internal carotid artery [ICA]), including intracranial aneurysms and stenosis in the anterior circulation.

Exclusion criteria: 1. Patients with contraindications to surgery, such as liver and kidney dysfunction. 2. Patients with a history of radial artery surgery (e.g., vascular access surgery for hemodialysis in uremia). 3. Acute cerebral infarction. 4. Peripheral vascular disease (subclavian artery stenosis or tortuosity). 5. TRA failed and converted to TFA.

### Data collection

Data collected included patient age, gender, underlying diseases, aortic arch type, whether the radial artery puncture or the form Simmon 2 catheter was routinely difficult, whether there was tortuosity in the brachiocephalic trunk, or in the C2–4 segment of the ICA, and surgical complications.

#### Transradial approach-related vascular access establishment surgical procedure

##### Radial artery puncture

After general anesthesia, the radial artery was punctured at the site of the strongest pulsation by using a Terumo puncture needle (Terumo, Japan). Upon successful puncture, a 6F radial artery sheath was inserted, and after this, a vasodilatory cocktail containing Nitroglycerine and Verapamil immediately injected into the sheath to prevent the spasm. During our procedure, we define more than three punctures or a duration exceeding 3 min as difficult to puncture.

#### The coaxial technique of the reformed Simmon 2 catheter combined with the intermediate catheter

The coaxial technique for a reformed Simmons 2 catheter, combined with an intermediate catheter, was performed as follows. A 260-cm 0.035onglidewire was advanced across the aortic arch into the descending aorta. The Simmons 2 catheter was then advanced over the wire until the secondary curve was positioned near the ascending aorta. The wire was retracted to just proximal to the secondary curve to increase support, and the system was advanced to allow the secondary curve to herniate into the ascending aorta, thereby reforming the reverse Simmons configuration for common carotid artery (CCA)/ICA selection.

To enhance stability, we applied a TRUST-style coaxial technique using an intermediate catheter (Jia Qi Bio, China). A 105-cm intermediate catheter was introduced coaxially over a 125-cm Simmons 2 catheter and guided into the descending aorta with the 0.035ntglidewire. After reforming the Simmons 2 catheter curve, the catheter tip was adjusted to engage the target CCA. Under roadmap guidance, the glidewire was advanced to the C3–C4 ICA, and the intermediate catheter was tracked to the C4 segment. Finally, the Simmons 2 catheter and glidewire were withdrawn.

#### Grouping method

Vascular access construction time was stratified into two groups using 15 min as a cutoff. The "easy" group included cases requiring less than 15 min, while the "difficult" group required more than 15 min.

### Statistical analysis

Statistical analyses were performed using Statistical Package for the Social Sciences 22.0 (IBM, Armonk, NY, USA). Normally distributed variables are presented as mean±standard deviation and compared using appropriate parametric tests; skewed variables are expressed as median (interquartile range) and compared using rank-sum tests. Categorical variables are expressed as n (%) and analyzed using the chi-square test. Variables with p<0.05 were entered into a multivariable binary logistic regression model.

## RESULTS

### General information

A total of 277 patients were included in the study, with 199 in the easy group and 78 in the difficult group. There were 143 males and 134 females. There were no statistically significant differences between the two groups in terms of hypertension, diabetes, hyperlipidemia, etc (all p>0.05). See Table 1.

**Table 1:** Univariate analysis results of patients undergoing neurointerventional vascular access construction via transradial approach.

Procedural data	Easy (n=199)	Difficult (n=78)	t/χ^2^	p
Male	103	41	0.0051	0.9431
Female	96	37		
Age	51.5±13.4	49.6±14.5	0.7147^t^	0.484
Hypertension	92	40	0.5731	0.449
Diabetes	88	37	0.2339	0.6287
Hyperlipidemia	83	36	0.4519	0.5014
Difficulty in radial artery puncture	34	30	14.412	<0.0001
Easy for radial artery puncture	165	48		
Type III aortic arch	157	63	0.1205	0.7285
Non-type III arch	42	15		
Difficulty in forming the Simmon 2 routinely	47	33	9.5295	0.0002
Form the Simmon 2 routinely	152	45		
Tortuosity of brachiocephalic trunk vessels	91	53	11.0842	0.0009
Straight brachiocephalic trunk vessels	108	25		
Sharp angle (<30°)	69	47	15.0677	<0.0001
Wide angle (>30°)	130	31		
Tortuosity in the C2–4 segment of the Target side internal carotid artery	73	50	10.7214	0.0011
Straight C2–4 segment of target side Internal carotid artery	126	28		
Surgical complications	41	19	3.24	0.0719
Radial artery spasm	35	16		
Radial artery occlusion	6	2		
Vascular perforation	0	1		

Note: Sharp angle (<30°) between brachiocephalic trunk vessels and target side common carotid artery; wide angle (>30°) between brachiocephalic trunk vessels and target side common carotid artery.

t Student's t-test.

### Univariate analysis results

There were statistically significant differences between the two groups in terms of difficulty in radial artery sheath insertion, whether the Simmon 2 catheter could be routinely formed, whether the brachiocephalic trunk vessels were tortuous, whether the angle between the brachiocephalic trunk vessels and the target side CCA was sharp (<30°), and whether there was tortuosity in the C2–4 segment of the ICA. There were no statistically significant differences between the two groups in terms of whether there was a type III arch (all p<0.05). See Table 1.

### Multivariate analysis results

Multivariate logistic regression analysis showed that difficulty in radial artery sheath insertion (OR 0.399, 95%CI 0.307–0.519, p<0.001), inability of the Simmon 2 catheter to routinely form (OR 0.393, 95%CI 0.300–0.515, p<0.001), tortuosity of the brachiocephalic trunk vessels (OR 0.337, 95%CI 0.246–0.463, p<0.001), sharp angle between the brachiocephalic trunk vessels and the target side CCA (<30°) (OR 0.357, 95%CI 0.265–0.479, p<0.001), and tortuosity in the C2–4 segment of the ICA (OR 0.384, 95%CI 0.257–0.470, p<0.001) were independent factors for difficulty in vascular access construction during neurointerventional therapy. See Table 2. The inverse OR values (OR<1) denote variables associated with an easier group, as the analysis was coded to prioritize "difficult group" as the primary endpoint.

**Table 2:** Multivariate logistic regression analysis results of patients undergoing neurointerventional vascular access construction via transradial approach.

Variables	B	SE	Wald	OR (95%CI)	p-value
Difficulty in radial artery puncture	-0.918	0.134	46.772	0.399 (0.307–0.519)	<0.001
Difficulty in forming the Simmon 2 catheter routinely	-0.934	0.138	45.699	0.393 (0.300–0.515)	<0.001
Tortuosity of brachiocephalic trunk vessels	-1.087	0.162	45.193	0.337 (0.246–0.463)	<0.001
Sharp angle (<30°) between Brachiocephalic trunk vessels and target side common carotid artery	-1.031	0.151	46.824	0.357 (0.265–0.479)	<0.001
Tortuosity in the C2–4 segment of target side internal carotid artery	-1.056	0.154	47.192	0.384 (0.257–0.470)	<0.001

CI: confidence interval; OR: odds ratio.

## DISCUSSION

The TRA is well established in coronary interventions^3^, but neurointerventionists generally have less experience with radial artery puncture. Limited proficiency, an incomplete learning curve, and weak radial pulsation in some patients may prolong puncture time and increase attempts. A failed initial puncture often leads to repeated attempts^4^, increasing radial artery stimulation, and the risk of spasm or occlusion^5^. Although ultrasound guidance can improve first-pass success^6^, achieving consistent puncture proficiency requires training and experience, remaining a key barrier to widespread TRA adoption in neurointervention.

TRA has been frequently reported for posterior circulation interventions because the vertebral artery connects directly to the subclavian artery, which can facilitate stable access construction^7^. In contrast, anterior circulation treatment via right TRA requires a stable working platform across the aortic arch and supra-aortic vessels. In this setting, the coaxial technique using a Simmons 2 catheter combined with an intermediate catheter represents the principal method for vascular access establishment. A key bottleneck is whether the Simmon2 configuration can be formed routinely. The conventional method advances the guidewire into the descending aorta to provide a fulcrum for controlled rotation and curve formation^7^. Our experience indicated that if the Simmon2 catheter failed to reach the descending aorta, super-selection of the target vessel could become unfeasible or markedly difficult.

We observed that certain aortic morphologies appeared to hinder Simmon 2 catheter formation. When the horizontal segment of the aortic arch is redundant and the descending aorta demonstrates a pronounced downward angulation, advancing the Simmon 2 catheter may drive the catheter deeply into the lower descending aorta. Such displacement reduces effective support and limits controlled rotation, thereby preventing formation of a stable reverse curve. Forming the catheter in the ascending aorta is technically possible but may increase the risk of arrhythmia and potential valvular injury; therefore, we avoided this technique. In formation-difficult cases, we switched to alternative shapes such as Simmon 1 and Vitek catheters, but this approach inevitably increased procedural time and fluoroscopy exposure. Importantly, there is currently no reliable approach to pre-evaluate Simmon 2 catheter formation feasibility solely from aortic arch morphology in routine practice. Consequently, the decision to persist with Simmon 2 catheter formation or to switch catheters often depends on operator experience. Future studies should develop imaging-based predictors that can guide early catheter selection and reduce trial-and-error during access construction.

**Figure 1:**
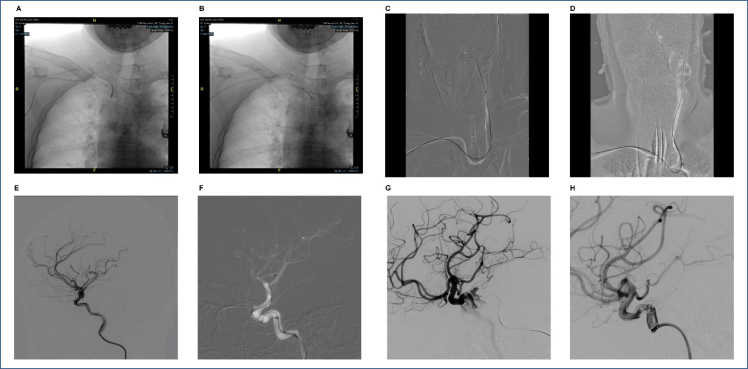
Representative angiographic examples of vascular access challenges and troubleshooting during transradial neurointerventional therapy. **(A)** The catheter was kinked at the subclavian artery; **(B)** Reverse rotation of the catheter was performed to restore the kinked segment; **(C)** The tortuous anatomy of the vessel elevated the tension on the device, thereby inducing deformation of the intermediate catheter; **(D)** Inter support with a V-18 guidewire was applied to improve the intermediate catheter stability; **(E)** The tortuosity of the internal carotid artery elevates the difficulty in placing the intermediate catheter to reach the C4 segment; **(F)** After the microcatheter reacheed M1 in Roadmap, the intermediate catheter was advanced through the tortuous segment of the internal carotid artery; **(G)** V-18 guidewire-induced of the internal carotid artery led to the carotid-cavernous fistula (CCF) during the intermediate-catheter delivery; **(H)** Following the deployment of embolization coils, the cavernous sinus fistula was completely not visualized.

Beyond the arch, tortuosity and angulation at the brachiocephalic trunk and subclavian artery can restrict the transmission of torque and forward force. When resistance is high, rotational force applied proximally may not translate smoothly to the catheter tip within the arch, and forcing advancement can result in catheter herniation or kinking. In one patient, kinking occurred during rotation due to proximal tortuosity; it was identified in time and corrected promptly, avoiding major consequences (Figures 1A, B). In such cases, we used a stiff guidewire to support the tortuous segment and maintain longitudinal tension, thereby stabilizing the catheter during rotation. Nevertheless, even when access could be constructed successfully, unfavorable proximal anatomy typically prolonged manipulation and increased fluoroscopy time, which were difficult to avoid.

Stability becomes particularly challenging during super-selection of the left CCA and its branches via right TRA. A prior report suggested that a sharp angle (<30°) between the brachiocephalic trunk, the aortic arch opening, and the left CCA can complicate catheter navigation^8^. Consistently, we found that a sharp angle (<30°) between the brachiocephalic trunk and the subclavian artery opening can also generate excessive system tension. Even if the Simmon 2 catheter is seated in the CCA, passing the sharp angle may produce recoil and instability, making it difficult to advance the intermediate catheter into the internal carotid arteries; this may further lead to herniation into the brachiocephalic trunk or the aortic arch^9^. Therefore, cautious advancement with continuous monitoring of tension and catheter configuration is essential. In our workflow, as the intermediate catheter approached the end of the C2–C3 segment, the Simmon 2 catheter was coaxially retracted while maintaining the 0.035-inch guidewire. Subsequently, a V-18 guidewire was introduced into the intermediate catheter to provide internal support, thereby improving system tension and stability (Figures 1C, D).

For definitive therapy, the intermediate catheter should be positioned as close to the lesion as feasible and maintained in a stable configuration to minimize displacement during stent or coil delivery^10^. However, marked tortuosity in the C2–C4 segment of the ICA may prevent smooth tracking because distal support is insufficient (Figure 1E). Excessive pushing is undesirable because it can provoke vasospasm and may precipitate dissection. Our strategy was to position the intermediate catheter at the proximal end of the tortuous segment, add V-18 internal support, and then super-select a microcatheter to the distal M1 segment of the middle cerebral artery. At that stage, the intermediate catheter was advanced coaxially across the tortuous segment (Figure 1F), while maintaining a stable distal microcatheter position to reduce the risk of perforation from unexpected displacement.

Notably, we encountered a serious wire-related complication: during rotation, the V-18 guidewire inadvertently displaced and perforated the cavernous segment. The event was recognized promptly and treated with coiling; postoperative oculomotor nerve palsy occurred without more severe consequences (Figures 1G, H). This case emphasized that internal support techniques can improve stability but require meticulous control of wire position, tip behavior, and rotation force, particularly in tortuous anatomy.

Several limitations of this study merit consideration. Primarily, it is a single-center retrospective study, which may limit the generalizability of the results. Furthermore, all neurointerventional therapy were performed by a single operator team, introducing potential operator-dependent variability. Finally, patients with failed TRA conversions to TFA were excluded, which may affect the overall estimation of TRA feasibility. In the future, more Prospective multicenter trials are needed to confirm our conclusions.

## CONCLUSION

Our preliminary experience indicated that difficulty in radial artery puncture and sheath insertion, inability to routinely form the Simmons 2 catheter, and tortuosity of the C2–C4 ICA—together with proximal supra-aortic tortuosity and sharp angulation (<30°)—were major contributors to difficult vascular access establishment during TRA neurointerventional therapy. These factors can compromise stability, prolong access construction, and increase fluoroscopy exposure, representing important barriers to broader adoption of TRA. Addressing these issues will require improved puncture proficiency (potentially supported by ultrasound guidance^6^), better preprocedural anatomical assessment to anticipate Simmon 2 formation difficulty, and continued refinement of catheter and wire-support strategies to enhance stability while minimizing complications.

## Data Availability

The datasets generated and/or analyzed during the current study are available from the corresponding author upon reasonable request.
